# Tumor-draining lymph node-derived tumor-specific memory CD8^+^ T cells: a key player in PD-1/PD-L1 immunotherapy

**DOI:** 10.1038/s41392-023-01356-0

**Published:** 2023-03-11

**Authors:** Enzhi Yin, Nan Sun, Jie He

**Affiliations:** 1 Department of Thoracic Surgery, National Cancer Center/National Clinical Research Center for Cancer/Cancer Hospital, Chinese Academy of Medical Sciences and Peking Union Medical College, Beijing, China; 2 State Key Laboratory of Molecular Oncology, National Cancer Center/National Clinical Research Center for Cancer/Cancer Hospital, Chinese Academy of Medical Sciences and Peking Union Medical College, Beijing, China

**Keywords:** Cancer microenvironment, Immunotherapy

A new study by Huang et al. in *Cell* identified the presence of tumor-specific memory CD8^+^ T cells in tumor-draining lymph nodes (TdLNs) and confirmed the critical role of this population in PD-1/PD-L1 immune checkpoint therapy for cancer.^1^ The work enriched our understanding of tumor-specific CD8^+^ T cell subsets and further refined the spatiotemporal mechanism of the antitumor effects of PD-1/PD-L1 immune checkpoint blockade (ICB).

PD-1 and PD-L1 ICB have made remarkable achievements in antitumor therapy. A traditional theory represents the major mechanism by which PD-1/PD-L1 ICB reactivates exhausted CD8^+^ T cells (T_EX_) in tumor microenvironment (TME), thereby restoring the autologous antitumor immunity.^2^ However, this theory cannot explain why only a portion of T_EX_ can response to PD-1/PD-L1 ICB and even “cold” tumors responses to ICB. More recently, accumulating studies point out that immune microenvironment of TdLNs may be a theoretical supplement for TME.^3^ At present, the study on the role of TdLNs in immunotherapy has just started. Huang’s work provides us with a new perspective on studying TdLNs.

Utilizing orthotopic and subcutaneous tumor mouse models, Huang et al. detected a group of memory CD8^+^ T cells in TdLNs that expressed TCF1^+^ TOX^-^ PD-1^low^. This group of identified CD8^+^ T cells was consistent in features of memory T cells, such as high expression of canonical memory-associated markers (CD127, CD122, and CD62L), semblable transcriptome and epigenome characteristics, as well as low expression of PD-1. Therefore, they named these cells tumor draining lymph node derived tumor-specific memory T cells (TdLN-T_TSM_). Significantly, compared with progenitor of exhausted T cells (T_PEX_) in TdLN and TME, T_TSM_ had a greater proliferation potential comparable to that of memory cells and exhibit an increased capability on antitumor.

Remarkably, authors next demonstrated that TdLN-T_TSM_ also existed in tumor draining lymph nodes from patients who suffered hepatocellular carcinoma. According to T cell receptor clonal comparison and pseudotime trajectory analysis, TdLN-T_TSM_ showed a developmental trajectory of T_PEX_ cells differentiated into tumor infiltrates and exhausted T cells, suggesting that TdLN-T_TSM_ may also play an important antitumor role in cancer patients.

To further investigate the antitumor effects of TdLN-T_TSM_, TdLN-T_TSM_ and other two tumor-specific CD8^+^ T cell subsets including TME-T_EX_ and T_PEX_ were adoptively transferred into tumor-bearing mice respectively. TdLN-T_TSM_ cells showed the most significant effect on inhibiting tumor growth.

Notably, TdLN-T_TSM_ was shown to be a key response cell for PD-1/PD-L1 ICB therapy compared with TdLN-T_PEX_ cells through both in vivo and in vitro experiments. Further, lymphadenectomy resulted in the failure of PD-L1 blocking antibody-mediated immunotherapy before or during PD-L1 ICB treatment. However, this effect was able to be restored by denovo transfusion of TdLN-T_TSM_ cells, which further verified that TdLN-T_TSM_ was a bona fide cell subgroup responding to PD-L1 ICB.

**Fig. 1:**
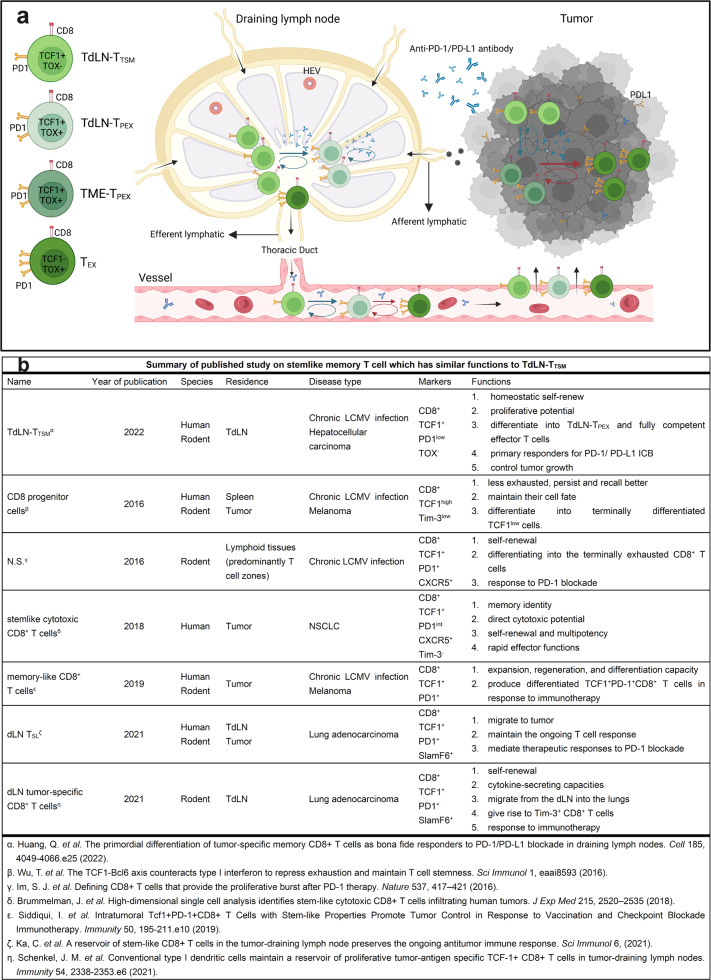
**a** The mechanism of tumor draining lymph nodes-tumor specific memory T cells (TdLN-T_TSM_) response to PD-1/PD-L1 immune checkpoint blocking. As a precursor of TdLN-progenitor of exhausted T cells (T_PEX_), T_TSM_ is located upstream of differentiation and persistently recruit various exhausted T cell subpopulation located in the tumor microenvironment (TME). The antitumor effect of PD-1/PD-L1 antibody is dependent on T_TSM_ cell subsets. The prerequisite for antitumor effect of PD-1/PD-L1 antibody is to first amplify T_TSM_ cell in draining lymph nodes, meanwhile promoting the differentiation into T_PEX_ which subsequently differentiate into exhausted CD8^+^ T cells (T_EX_). Finally, the progeny of these cells enters TME through peripheral circulation and plays an antitumor role. Created with BioRender.com. **b** A summary of published studies on stemlike memory CD8^+^ T cells which have similar functions to TdLN-T_TSM_. TdLN-T_TSM_ tumour-draining lymph nodes-tumor specific memory T cells, N.S. nothing special, T_SL_ stem-like T cells, LCMV lymphocytic choriomeningitis virus, NSCLC non-small cell lung cancer, T_PEX_ progenitor of exhausted T cells, ICB immune checkpoint blockade

In summary, these data established the presence and role of the TdLN-T_TSM_ in generating primary antitumor immune responses following anti-PD-1/PD-L1 ICB, besides providing insights into the spatio-temporal mechanism of PD-1/PD-L1 ICB against tumor (Fig. 1a). Importantly, this work led us to profound reflection.

First, to dig into stemlike memory CD8^+^ T cells, we summarized previous literature that confirmed the presence of intra or extra-tumoral stemlike memory T cell which had broadly similar functions to TdLN-T_TSM_ (Fig. 1b). In contrast to cells from other research, TdLN-T_TSM_ were shown to be free of exhaustion associated epigenetic scars, suggesting this population was a steady resident in TdLN. It is well known that tumor escape caused by T cell exhaustion is the main cause limiting the efficacy of chimeric antigen receptor (CAR) T cell therapy.^4^ This study implied that TdLN-T_TSM_ may be a promising candidate of CAR T cell therapy, correspondingly, TdLN which removed by surgery may become a more suitable source of CAR T cell in place of peripheral blood (PB).

Next, as a bona fide cell responding to PD-1/PD-L1 ICB, proportion of T_TSM_ in TdLN may be a novel predictable biomarker of effect after ICB to enable identification of patients in need of alternative treatment strategies. Future studies should focus on confirming the particular marker and existence of T_TSM_ on various tumor types, as well as investigating a potential role in progression and outcome of different pathological types and stages. Moreover, the percentage of T_TSM_ in TdLN, non-TdLN and PB should also be detected in future work.

More importantly, based on the impressive data from adoptive transfer study of TdLN-T_TSM_ in animal model, TdLN-T_TSM_ was a potential choice of adjuvant or combination therapy. Clinical application of immunotherapy by utilizing this population from TdLN should be more explored to potentiate ICB therapy in a real-world setting.

Last not least, this work led us to a renewed look at the effect of TdLN in oncological surgery. To prevent the spread of tumor cells in patients undergoing surgical treatment, complete lymphadenectomy has been well accepted as a traditional clinical practice. However, assisted by the development of ICB therapy, especially PD-1/PD-L1 ICB, reassessment of optimal strategy of lymphadenectomy is clearly warranted. A study from Fear et al. indicated that LN resection may impair the efficacy of adjuvant immunotherapy in a pre-clinical model.^5^ According to Huang’s research, we inferred leaving tumor-free lymph nodes intact during operation may enable patients to benefit from PD-1/PD-L1 ICB therapy. Further study needs to be performed to verify speculation mentioned above.
